# Multicenter Cross-Sectional Study of Oral Health and Hygiene Practices Among Pregnant Women

**DOI:** 10.3390/jcm13237315

**Published:** 2024-12-02

**Authors:** Roberto Lo Giudice, Canio Martinelli, Angela Alibrandi, Alessandro Mondo, Renato Venezia, Maria Grazia Cannarozzo, Francesco Puleio, Raffaella Pollicino, Giuseppe Lo Giudice, Antonio Simone Laganà

**Affiliations:** 1 Department of Human Pathology of Adults and Developmental Age, Messina University, 98100 Messina, Italy; roberto.logiudice@unime.it; 2 Unit of Gynecology and Obstetrics, Department of Human Pathology of Adult and Childhood “G. Barresi”, Messina University, 98122 Messina, Italy; caniomartinelli.md@gmail.com (C.M.); alessandromondo391@gmail.com (A.M.); 3 Sbarro Institute for Cancer Research and Molecular Medicine and Center for Biotechnology, College of Science and Technology, Temple University, Philadelphia, PA 1912, USA; 4 Department of Economics, Messina University, 98122 Messina, Italy; aalibrandi@unime.it; 5 Department of Maternal and Child Health and Urological Sciences, Sapienza University of Rome, Viale del Policlinico, 155, 00161 Rome, Italy; 6 Unit of Obstetrics and Gynecology, “Paolo Giaccone” Hospital, Department of Health Promotion, Mother and Child Care, Internal Medicine and Medical Specialties (PROMISE), University of Palermo, 90127 Palermo, Italy; renato.venezia@unipa.it (R.V.); antoniosimone.lagana@unipa.it (A.S.L.); 7 Independent Researcher, 95100 Catania, Italy; mg.cannarozzo@alice.it; 8 Department of Biomedical and Dental Sciences and Morphofunctional Imaging, Messina University, 98122 Messina, Italy; pollicinoraffaella@hotmail.com (R.P.); logiudice.g@unime.it (G.L.G.)

**Keywords:** oral health, pregnancy, DMFT, oral hygiene practices

## Abstract

**Background**: Hormonal, vascular, and behavioral changes during pregnancy can negatively impact a woman’s oral health, resulting in conditions such as gingivitis, dental sensitivity, and caries. Although oral health is critical during pregnancy, it remains frequently overlooked. In this study, the oral health status and hygiene practices of pregnant women attending two university hospitals in southern Italy were evaluated. **Methods**: A cross-sectional cohort study was conducted with 72 pregnant women. Data were collected through an anonymous questionnaire that evaluated oral hygiene habits, dental visits, and oral pathologies, followed by a clinical examination. The examination included the assessment of the Decayed, Missing, Filled Teeth (DMFT) index and the Oral Hygiene Index (OHI) scores. **Results**: Among the participants, 61% were in their 9th month of pregnancy. The mean DMFT score was 7.9, and the mean OHI score was 3.6. Only 19.4% of participants had visited a dentist in the past 6 months, while 97.2% reported brushing their teeth just once per day. Gingival bleeding was reported by 72.2% of the women, and 38.9% experienced dental sensitivity, with both conditions worsening during pregnancy. **Conclusions**: The need for greater oral hygiene education and regular dental care during pregnancy is highlighted in this study. Integrating oral health maintenance into prenatal care programs is essential for preventing pregnancy-related oral diseases and promoting maternal and fetal well-being.

## 1. Introduction

During pregnancy, a woman’s body undergoes significant transformations driven by a complex interplay of hormonal, genetic, and environmental factors to support fetal development [1]. The main hormones involved include estrogens, progesterone, follicle-stimulating hormone, luteinizing hormone, and chorionic gonadotropin [1]. Estrogens (estrone, estradiol, and estriol) are secreted by the corpus luteum during the first trimester and by the placenta in subsequent trimesters; their levels increase drastically, causing the expansion of the uterus, the enlargement of the genitals and breasts in preparation for milk production, and inducing the relaxation of pelvic ligaments, making the sacroiliac joints and pubic symphysis more elastic in preparation for childbirth [2]. Progesterone, which increases during pregnancy, maintains the uterine wall thickness, prevents miscarriage, and contributes to symptoms such as fatigue, drowsiness, and fluid retention [3]. Follicle-stimulating hormone (FSH) and luteinizing hormone (LH), which regulate the menstrual cycle, decrease to prevent ovulation [4]. The pregnancy hormone chorionic gonadotropin (HCG), produced by the placenta, stimulates the corpus luteum to secrete large amounts of estrogen and progesterone, ensuring the endometrium continues to develop and store nutrients, preventing its breakdown, which typically occurs during menstruation [5,6]. These hormonal fluctuations affect a woman’s metabolism by increasing caloric needs and contributing to fluid retention, leading to physical changes such as swelling, weight gain, fatigue, and emotional changes like mood swings and nausea, commonly known as “morning sickness” [7].

The physiological and behavioral changes occurring during pregnancy can negatively affect oral health [8,9,10]. Hormonal, vascular, and immunological alterations often cause gingival tissues to exhibit an exaggerated inflammatory response to local inflammatory factors such as dental plaque, tartar, or overhanging restorations or prostheses, leading to edema, enlargement, and bleeding, which can evolve into specific periodontal conditions (gingivitis, periodontitis, and pregnancy epulis) that tend to regress after pregnancy [8,11]. Gingivitis affects 30–75% of pregnant women, while pregnancy epulis occurs in up to 10% [12,13]. Elevated levels of progesterone and estrogen lead to increased salivary secretions, influencing the quantity and quality of saliva, oral bacterial flora, and sensitivity to carbohydrates [14,15,16]. It is common for pregnant women to experience hypersalivation, which is generally temporary and improves after childbirth [16]. Progesterone also alters the composition of saliva, increasing compounds such as bicarbonate and reducing the presence of starch, making the saliva more viscous and lowering its pH [14,15,16,17]. This effect reduces saliva’s buffering capacity, increasing the risk of caries and causing greater sensitivity to tastes and odors [14,17,18,19]. The sudden rise in estrogen, progesterone, and chorionic gonadotropin, physiological in pregnancy, also induces nausea and vomiting [20,21]. About 80% of pregnant women experience nausea in varying degrees and frequency [20,21]. In 1% of cases, hyperemesis gravidarum can occur, characterized by severe nausea and vomiting, leading to weight loss, ketosis, dehydration, and electrolyte imbalances, requiring hospitalization for nutrition and intravenous fluids [20,21]. These episodes, if not properly managed with pH-neutralizing techniques and avoiding brushing teeth immediately after vomiting, can lead to enamel erosion, increasing the risk of caries and dental sensitivity [22,23].

During pregnancy, hormonal fluctuations, particularly increased levels of estrogen and progesterone, play a pivotal role in influencing oral health. These hormonal changes are associated with an exaggerated inflammatory response, leading to conditions such as pregnancy gingivitis and periodontitis. Furthermore, modifications in salivary flow and composition can exacerbate oral health challenges. Addressing these changes is critical, as maternal oral health is not only vital for the well-being of the mother but also has implications for fetal health, with poor oral health being linked to adverse pregnancy outcomes, such as preterm birth and low birth weight. These findings highlight the necessity of early intervention and customized dental care plans for pregnant women.

The aim of this multicenter cross-sectional cohort study was to evaluate the oral health and hygiene practices among pregnant women.

## 2. Materials and Methods

### 2.1. Study Design

This research is a multicenter cross-sectional study. It was conducted as a cross-sectional study to assess the relationship between exposure to risk factors and the occurrence of oral health conditions in pregnant women at a single time point. This design was chosen to capture a snapshot of oral health and its potential correlates during pregnancy.

An anonymous questionnaire was developed by a researcher from Messina University and administered to pregnant women at the University Hospitals Policlinico G. Martino and Policlinico “Paolo Giaccone”, involving all patients attending the gynecology departments of these hospitals. The questionnaire was written in Italian and completed anonymously by patients. The study design, data collection, analysis, and interpretation adhered to the Strengthening the Reporting of Observational Studies in Epidemiology (STROBE) guidelines for observational research. This study was conducted in compliance with the Declaration of Helsinki, and participants were provided with a brief written description of the study’s objectives and privacy regulations regarding data collection. Written informed consent was obtained from all subjects before their participation. Participants were thoroughly informed about the research goals, the procedures involved, and the potential benefits. They were assured of complete anonymity in the publication of results, with the information collected being used solely for scientific purposes. Each participant had the opportunity to ask questions and withdraw from this study at any point without any consequences. Upon completion, the questionnaires were handed to the staff. Refusal to complete the questionnaire did not affect the medical care received. The completed questionnaires were then given to a third party for result analysis. The study protocol was approved by the Ethics Committee “Comitato Etico Locale Palermo 1” of the “Paolo Giaccone” University Hospital (protocol n. 04/23 of 02/11/2023) considering the non-invasive nature of tests carried out.

Inclusion Criteria: pregnant patients attending the University Hospital Policlinico G. Martino or the University Hospital Policlinico “Paolo Giaccone”.

Exclusion Criteria: patients with dental diseases (e.g., hypocalcification, hypoplasia, fluorosis, and molar-incisor hypomineralization syndrome) or other oral pathologies.

### 2.2. Description of the Questionnaire

The questionnaire comprised the following four sections:

- The first section collected general demographic and obstetric data, such as age, number of pregnancies, and the gestational month of the current pregnancy.
- The second section assessed oral hygiene habits before and during pregnancy, including the timing of the last dental visit, daily brushing frequency, type of toothbrush bristles used (soft, medium, or hard), and the use of dental floss and mouthwash.
- The third section explored oral pathologies, including gingival bleeding (spontaneous or induced), its onset (before or during pregnancy), and whether it worsened during pregnancy. It also addressed dental sensitivity, its onset, and progression during pregnancy. Additional questions examined vomiting episodes and oral hygiene practices afterward, as well as changes in carbohydrate consumption during the day and at night, including subsequent toothbrushing habits. Additionally, this section asked the patient to describe their toothbrushing technique to assess whether it was performed correctly.
- The fourth section focused on the following behavioral changes related to pregnancy: presence of vomiting and whether the patient brushed their teeth afterward; increased carbohydrate intake; night-time carbohydrate intake and whether the patient brushed their teeth afterward.

After completing the questionnaire, an experienced and trained dentist performed a clinical examination of the patient’s oral cavity. The patient’s oral hygiene was subjectively assessed, determining whether they had good, average, or poor hygiene based on the presence of plaque, tartar, and gingival inflammation.

For each patient, the Decayed, Missing, Filled Teeth (DMFT) index and an Oral Hygiene index (OHI) were recorded, derived from the sum of various parameters. For the OHI index, each parameter was assigned a score according to the following methodology:

- Last dental visit:
  - <6 months = 1 point
  - 6 months = 0 points
  - 18 months = −1 point;
- Number of times the patient brushes their teeth daily:
  - 0 times = −1 point
  - 1 time = 0 points
  - 2 times = 1 point
  - 3 or more times = 2 points;
- Correct/incorrect brushing technique:
  - Correct = 1 point
  - Incorrect = −1 point;
- Toothbrush bristle type:
  - Soft = 1 point
  - Medium = 0 points
  - Hard = −1 point;
- Use of dental floss:
  - Yes = 1 point
  - No = 0 points
- Use of mouthwash:
  - Yes = 1 point
  - No = 0 points;
- The OHI score was then categorized as follows:
  - Score ≥5: Excellent oral hygiene
  - Score 3–4: Good oral hygiene
  - Score 0–2: Average oral hygiene
  - Score ≤−1: Poor oral hygiene.

### 2.3. Statistical Analysis

A descriptive analysis of the sample characteristics and oral hygiene habits of the patients included in this research was conducted. The following variables were analyzed:

- Age;
- Number of pregnancies;
- Date of the last dental visit: less than 6 months, more than 6 months, more than 1 year, or never;
- Number of times the teeth are brushed daily;
- Brushing technique: correct or incorrect;
- Toothbrush bristle type: soft, medium, or hard;
- Presence of gingival bleeding: spontaneous or induced, onset before or during pregnancy, and whether it increased during pregnancy;
- Presence of dental sensitivity: onset before or during pregnancy, and whether it increased during pregnancy;
- Use of dental floss;
- Use of mouthwash;
- Presence of vomiting;
- Brushing teeth after vomiting;
- Carbohydrate intake: increased, decreased, or unchanged;
- Night-time carbohydrate intake and whether teeth are brushed afterward;
- DMFT and OHI.

The variables analyzed were compared with DMFT and OHI using Spearman’s Rho coefficient to identify correlations.

Pearson’s Chi-square test was applied to evaluate the degree of association between the “oral hygiene level” variable and all other variables.

To determine the adequacy of the sample size, a power analysis was conducted. The parameters used for this analysis included an expected medium effect size (Cohen’s d = 0.5), a significance level (alpha) of 0.05, and a desired power of 0.85. The power analysis indicated that a sample size of approximately 72 participants would be sufficient to detect statistically significant differences with these parameters.

## 3. Results

The questionnaire was completed by 72 pregnant women, 34 from the University Hospital Policlinico G. Martino in Messina and 38 from the University Hospital Policlinico “Paolo Giaccone” in Palermo. All 72 participants completed the questionnaire, and no patients refused to fill it out. No patients were excluded from this study due to the exclusion criteria.

### 3.1. Descriptive Analysis of the Sample

In the study cohort, 13% of participants were in their 4th month of pregnancy, 17% in their 7th month, 33% in their 8th month, and 61% in their 9th month. The mean age of the participants was 31 years, ranging from 20 to 44 years. The recorded mean DMFT index was 7.9 ± 4.5, while the mean OHI index was 3.6 ± 1.9.

**Figure 1:**
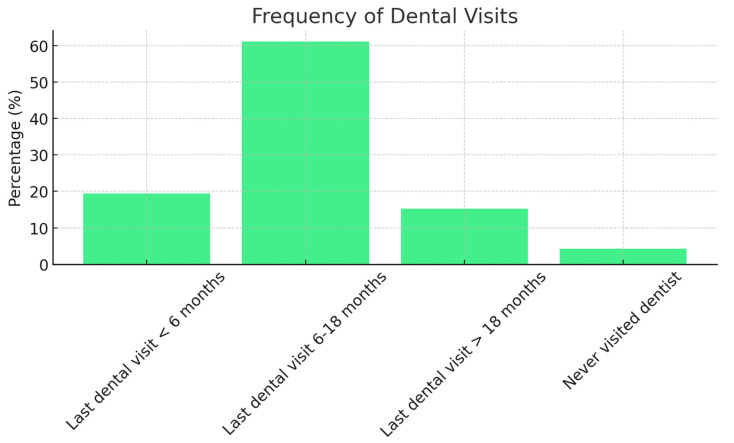
Distribution of dental visit frequency among study participants.

Regarding the date of the last dental visit, 19.4% of the participants had consulted a dentist within the past 6 months, 61.1% between 6 and 18 months ago, 15.3% over 18 months ago, and 4.2% reported never having visited a dentist (Figure 1).

Concerning oral hygiene, 97.2% of participants brushed their teeth at least once daily, with 58.3% brushing twice, 26.4% brushing three times, and 5.6% brushing four times per day.

**Figure 2:**
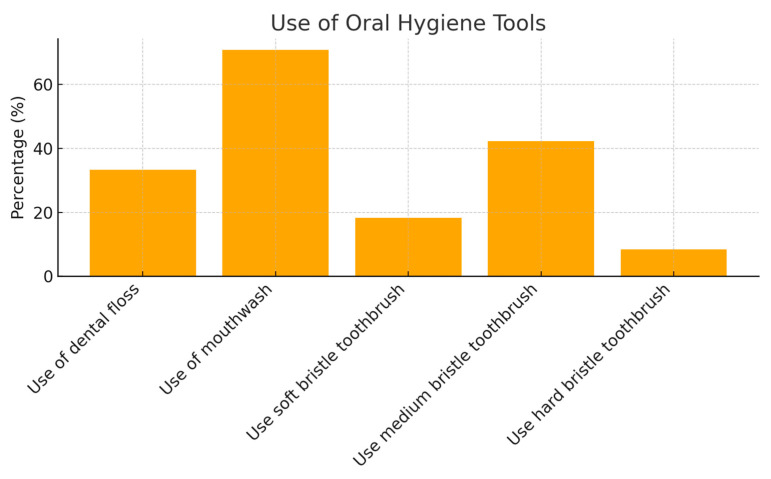
Use of oral hygiene tools.

Regarding the use of additional hygiene tools, 33.3% of the sample reported using dental floss, and 70.8% reported using mouthwash as part of their oral hygiene routine (Figure 2).

In terms of toothbrush type, 18.3% of the sample used a toothbrush with soft bristles, 42.2% used medium bristles, 8.4% used hard bristles, and 30.9% were unaware of the type of bristles used.

Regarding gingival bleeding, 72.2% of the sample reported experiencing it, with 90.4% reporting that it was induced by oral hygiene procedures, and 9.6% reporting spontaneous bleeding. Among those who reported gingival bleeding, 32.5% stated that the bleeding began with the onset of pregnancy, while 67.5% reported that it was present before pregnancy. Furthermore, 41.7% of women reported that their gingival bleeding increased during pregnancy.

Regarding dental sensitivity, 38.9% of the sample reported experiencing it, with 10.7% stating they had experienced sensitivity before pregnancy, 14.3% reporting that it began during pregnancy, and 75% reporting that it was present both before and during pregnancy. Among the women with dental sensitivity, 61.9% reported that sensitivity increased during pregnancy, while 38.1% stated it remained unchanged.

Regarding vomiting episodes, 61.1% of women reported experiencing nausea or vomiting during pregnancy, but 48.9% of them did not brush their teeth afterward.

Concerning night-time carbohydrate intake, 22.2% of women reported an increase in carbohydrate consumption during the night, and of those, 88.8% did not brush their teeth after consuming carbohydrates at night.

**Figure 3:**
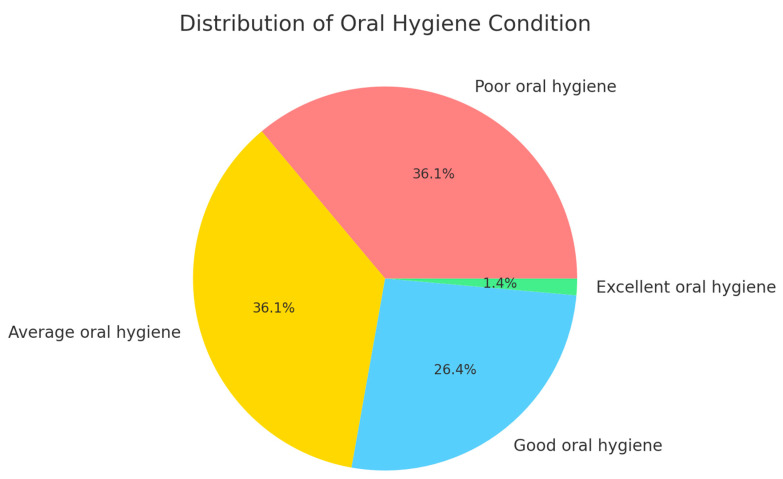
Distribution of oral hygiene levels among participants based on clinical evaluation.

The objective clinical examination revealed that 36.1% of the patients had poor oral hygiene, 36.1% had average oral hygiene, 26.4% had good oral hygiene, and 1.4% had excellent oral hygiene (Figure 3).

**Figure 4:**
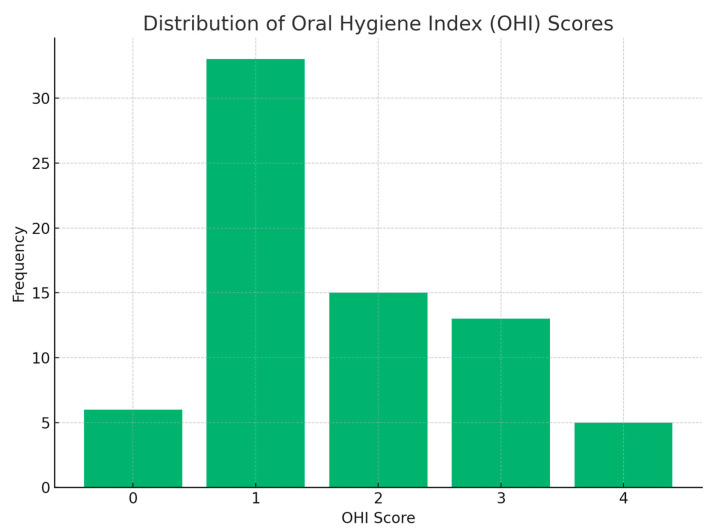
Distribution of OHI score.

The distribution of the Oral Hygiene Index (OHI) scores among the study participants. The OHI score, calculated based on brushing frequency, use of dental floss, and use of mouthwash, ranged from 0 to 4. The data reveal that a significant portion of the participants, approximately 40%, scored an OHI of 2, indicating an average level of oral hygiene. Around 30% achieved a score of 3, reflecting good oral hygiene practices. Conversely, about 20% of the participants had a score of 1, suggesting a need for improvement in their oral hygiene routine. Only a small percentage, roughly 10%, reached the highest score of 4, demonstrating excellent oral hygiene habits. This distribution highlights the variation in oral hygiene practices among the pregnant women surveyed and suggests areas for potential improvement in oral health education and care (Figure 4).

### 3.2. Statistical Results

Spearman’s Rho coefficient revealed that the OHI was negatively correlated with the variables “date of last dental visit” (*p* < 0.001) and “brushing technique” (*p* < 0.001), and that the DMFT was negatively correlated with the variable “oral hygiene level” (*p* < 0.05). A positive correlation was also found between OHI and the variables “use of dental floss” (*p* < 0.001), “use of mouthwash” (*p* < 0.001), and “oral hygiene level” (*p* < 0.001).

Furthermore, a negative correlation was observed between the variables “vomiting” and “number of times teeth are brushed daily”.

**Figure 5:**
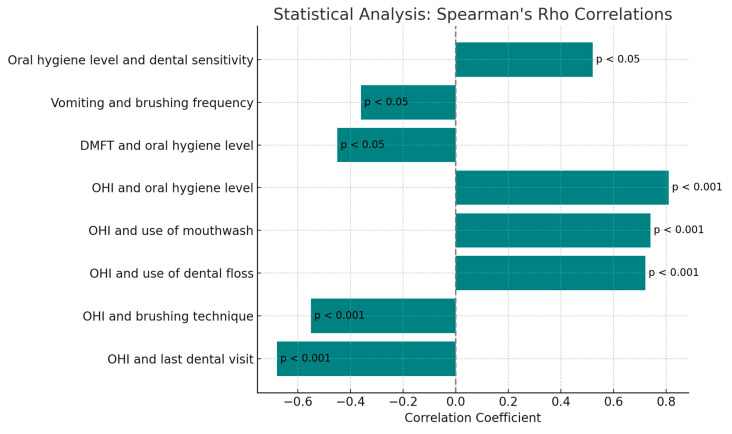
Spearman’s analysis.

Pearson’s Chi-square test demonstrated a statistically significant association between “oral hygiene level” and “dental sensitivity” (Figure 5).

## 4. Discussion

In this study, the aim was to explore the oral health of pregnant women attending two university hospitals in southern Italy. The results gathered through an anonymous questionnaire provide an overview of the oral conditions of pregnant women, offering epidemiological data, oral hygiene habits, and an assessment of oral health status [24,25,26]. The DMFT index used in this research provides a quantitative measure of dental caries and dental treatment; the DMFT index counts the number of decayed, missing due to caries, and filled teeth in an individual. A lower DMFT score indicates better dental health, while a higher score suggests a higher prevalence of dental problems. To gain insight into the oral hygiene habits of the sample, the OHI index was developed. Hormonal, vascular, and behavioral changes occurring during pregnancy can negatively influence oral hygiene habits [8,9,10]. Therefore, this index may be more useful for research purposes than the DMFT index.

Most participants in this study (61%) were in their ninth month of pregnancy, with a mean age of 31 years. Only 19.4% of the sample had undergone a dental visit within the last 6 months. A negative correlation was found between OHI and the date of the last dental visit, suggesting that patients who had not seen a dentist recently exhibited worse oral hygiene habits. The data show that the majority of the patients (58.3%) brushed their teeth twice a day, while 26.4% did so three times a day. However, it is concerning that 97.2% of the women brushed their teeth only once a day. This behavior may be attributed to various factors related to pregnancy, such as fatigue and nausea, which can reduce motivation to maintain optimal oral hygiene. [9,27,28] A recent meta-analysis demonstrated that brushing less than twice a day is associated with a lower oral health-related quality of life (OHRQoL) in pregnant women [29]. Brushing at least twice a day has been shown to lead to better oral hygiene maintenance, improved OHRQoL, and better plaque control [30,31]. Moreover, 54.2% of the sample used an incorrect brushing technique. Only 33.3% of the patients regularly used dental floss, while 70.8% used mouthwash, indicating incomplete oral hygiene practices [32]. Dental flossing, as an adjunct to toothbrushing, enhances these benefits [33]. An incorrect brushing technique and lack of dental floss use are factors that can lead to insufficient oral hygiene, contributing to gingival inflammation [34]. The use of dental floss, mouthwash, and the overall oral hygiene level were positively correlated with OHI; patients who used dental floss and mouthwash and had better oral hygiene habits tended to have higher OHI scores.

An interesting finding concerns the choice of toothbrush. where only 18.3% used a soft-bristled toothbrush, which is recommended by professionals to prevent gum and enamel damage [35]. The prevalence of medium-bristled (42.2%) and hard-bristled (8.4%) toothbrushes suggests a need for more education on safe oral hygiene practices during pregnancy. Gingival bleeding was reported by 72.2% of patients, with most (90.4%) experiencing it during oral hygiene procedures. Furthermore, 41.7% of women reported that gingival bleeding increased during pregnancy, underscoring the significant impact of physiological changes on oral health. This finding is consistent with the literature, which highlights an increase in gingivitis during pregnancy due to hormonal fluctuations [17,36,37,38].

Dental sensitivity was reported by 38.9% of patients, with 61.9% stating that their sensitivity increased during pregnancy. Pearson’s Chi-square test demonstrated a statistically significant association between oral hygiene level and dental sensitivity, showing that pregnant women with poorer oral hygiene are more likely to develop dental sensitivity than those with better oral hygiene levels.

Among women who experienced vomiting (61.1%), 51.1% brushed their teeth after vomiting. Although dental erosion due to vomiting is typically observed in patients with chronic vomiting over several years, its occurrence depends on the severity and frequency of the episodes, as well as oral hygiene habits following exposure to gastric acid. Additional factors such as the mineralization of dental hard tissues and the amount and quality of secreted saliva also play a role [39,40]. Patients should be instructed to rinse their mouths with a pH-neutralizing solution or plain water instead of brushing their teeth immediately after vomiting. Gastric acids, with a pH of 1.5, have a severe impact on teeth, so patients should be encouraged to delay tooth brushing for at least one hour after vomiting [40]. Additionally, patients at risk for dental erosion should always use a fluoride source, such as toothpaste and/or rinse containing fluoride combined with stannous ions [41,42]. Dentists should instruct patients on the importance of using a tongue cleaner to remove acidic residue that accumulates on the papillae after vomiting [43].

Regarding night-time carbohydrate intake, 22.2% of women reported consuming carbohydrates at night; alarmingly, 88.8% of them did not brush their teeth afterward. Nocturnal eating has been identified as a significant predictor of missing teeth, periodontal disease, and active caries [44]. Only 26.4% of the sample examined showed good oral hygiene during the objective examination, with 1.4% exhibiting excellent hygiene. This finding suggests that most pregnant women do not pay sufficient attention to oral hygiene. Spearman’s Rho coefficient indicated that DMFT was negatively correlated with the “oral hygiene level” variable (*p* < 0.05), meaning that a higher DMFT score was associated with poorer oral hygiene. The OHI and DMFT scores provide valuable insights into the oral health status of the study population. The correlation observed between poor OHI scores and higher DMFT values indicates that inadequate oral hygiene is strongly associated with increased caries experience. This correlation underscores the clinical importance of promoting oral hygiene education and preventive measures, particularly in pregnant women, as improved oral hygiene could significantly reduce caries prevalence and enhance overall oral health during pregnancy. Pregnant women with a higher DMFT index, indicative of poorer dental health, tend to neglect oral hygiene during pregnancy more than those with a lower DMFT index. This information may help clinicians predict the evolution of oral hygiene in pregnant women, allowing them to be included in appropriate oral hygiene maintenance protocols.

A review of the literature examined the oral health status of non-pregnant women using the DMFT index. One study reported a high mean DMFT value of 12.23 ± 6.37 among fertile women aged 20 to 45 years, highlighting significant dental caries and restorative requirements within this population [45]. In the second study, the mean DMFT for postpartum women was 8, suggesting a relatively better oral health status compared to other groups [46]. The third study found no significant differences in DMFT scores between women with preterm birth and those without, implying that dental caries may not directly influence preterm birth outcomes [47].

A comparison of these data reveals that the mean DMFT index observed in our study among pregnant women was significantly higher than that reported for non-pregnant women. The first referenced study reported a mean DMFT of 12.23 ± 6.37 for fertile women, while in our pregnant cohort, the DMFT was around 15.2, indicating increased dental issues likely due to hormonal changes and decreased dental care during pregnancy and reduced oral hygiene practices [45]. The second study found a lower DMFT of 8 among postpartum women (age 30–39), which may reflect improvements in oral care post-pregnancy [46]. The third study showed no significant differences in DMFT between women with and without preterm birth, with DMFT scores around 10.5, suggesting that preterm birth does not influence dental caries risk directly [47]. Specifically, our study showed that younger pregnant women (ages 20–29) had a higher DMFT score compared to non-pregnant women of the same age group. This contrast is especially evident when comparing the postpartum group’s mean DMFT of 7.9, reflecting better dental health outcomes post-pregnancy.

This comparison underscores the need for targeted dental care interventions during pregnancy to reduce the risk of dental caries and improve overall oral health outcomes.

The strength points of this research include its multicenter design, which enhances the generalizability of the findings by involving pregnant women from two university hospitals. This study also provides valuable insights into the oral health habits and conditions of pregnant women, an area often overlooked in general healthcare. The use of both subjective (questionnaire) and objective (clinical examination) methods to assess oral hygiene adds robustness to this study. Additionally, the development of the Oral Hygiene Index (OHI) offers a novel, more sensitive tool for evaluating oral hygiene behaviors compared to traditional caries indices like DMFT.

Our results are consistent with those of Togoo et al., who reported a general lack of awareness among pregnant women regarding pregnancy gingivitis, including its causes, effects, treatments, and preventive measures [8]. Additionally, many participants expressed the need for oral health education before the onset of pregnancy; Togoo et al. concluded that collaborative efforts among general dentists, pediatric dentists, public health dentists, and medical professionals are essential to increase awareness among pregnant women regarding the importance of timely dental care to prevent adverse pregnancy-related outcomes caused by gingivitis and periodontitis [8].

Similarly, the systematic review by Tenenbaum and Azogui-Levy observed that many pregnant women demonstrated limited knowledge and awareness of oral health and its implications during pregnancy [10]. The authors emphasized the importance of targeted educational interventions to improve oral health literacy and practices in this population. Our findings align with these conclusions, as we identified a significant lack of awareness among pregnant women regarding oral health issues, particularly pregnancy gingivitis. This underscores the critical need for structured educational programs and collaborative initiatives involving healthcare professionals to improve maternal oral health and prevent potential complications during pregnancy.

### Limitations

A key limitation of this study lies in its reliance on self-reported data, which may be influenced by biases such as recall errors and social desirability, potentially compromising the accuracy of responses. While the questionnaire used was based on established instruments in maternal and oral health research, it was not formally validated prior to this study. To address this limitation, we recommend that future research include a pilot study to validate the questionnaire, ensuring it accurately measures the intended variables and aligns with international standards for oral health assessments.

Another limitation is the reliance on self-reported oral hygiene behaviors, such as brushing and flossing frequency, which are susceptible to overreporting due to social desirability bias. This could affect the reliability of the findings regarding hygiene practices. Future studies should incorporate objective measures, such as clinical evaluations or plaque indices, to validate self-reported behaviors and provide more robust data.

Although this study recognizes an increase in carbohydrate intake among pregnant women, it does not include a detailed dietary analysis, limiting the understanding of nutritional influences on oral health. Diet plays a critical role in oral health, and a more comprehensive assessment of dietary patterns would have provided additional insights into the relationship between nutrition and oral health outcomes. Future research should include detailed dietary evaluations to better understand how specific nutritional behaviors influence oral health during pregnancy.

Furthermore, the absence of a control group comprising non-pregnant women limits the ability to isolate and evaluate the specific impacts of pregnancy on oral health outcomes. Including a control group in future studies would allow for more robust comparisons and a clearer understanding of the effects of pregnancy-related hormonal and physiological changes on oral health.

Finally, the sample was geographically limited to a specific region in southern Italy, which may reduce the external validity of the findings for other populations. Future studies should aim to include more diverse populations to improve the generalizability of the results.

## 5. Conclusions

In this study, the unique challenges pregnant women encounter in maintaining oral health are highlighted and the key areas for medical intervention and oral hygiene education are identified. In this study, the critical need for targeted educational interventions to enhance the oral health of pregnant women is underscored. The increased dental care needs of women during pregnancy are also emphasized. Lastly, the management of oral health in pregnant women should involve a multidisciplinary team of healthcare professionals, with dentists playing a pivotal role in preventive care and education.

## Data Availability

Data are available upon request by the corresponding author.
